# The preventive effect of sodium-glucose co-transporter-2 inhibitors on atrial fibrillation and atrial flutter in patients with chronic kidney disease: a meta-analysis

**DOI:** 10.3389/fphar.2025.1585491

**Published:** 2025-05-20

**Authors:** Qian Yang, Chen Wang, Wenjing Wang, Yanhong Li, Yi Dang

**Affiliations:** ^1^ Department of Cardiology, Hebei General Hospital, Shijiazhuang, Hebei, China; ^2^ Department of Internal Medicine, Graduate School of Hebei Medical University, Shijiazhuang, Hebei, China

**Keywords:** chronic kidney disease (CKD), sodium-glucose co-transporter-2 inhibitors (SGLT2i), atrial fibrillation, atrial flutter, meta-analysis

## Abstract

**Background:**

The presence of atrial fibrillation (AF) and atrial flutter (AFL) in patients with chronic kidney disease (CKD) can exacerbate renal dysfunction, which in turn increases the onset of AF or AFL. Sodium-glucose co-transporter-2 inhibitors (SGLT2i) have been proven to have cardiac and renal protective effects. The meta-analysis was performed to investigate whether SGLT2i can reduce the risk of AF/AFL in patients with CKD.

**Methods:**

PubMed, Embase, Cochrane Library, and Clinical Trials.gov were searched up to December 2024. Randomized controlled trials (RCTs) comparing of SGLT2i and placebo on AF/AFL in patients with CKD were included. Risk ratio (RR) with 95% confidence interval (CI) were calculated in the overall population and selected subgroups.

**Results:**

10 RCTs involving 28,712 patients were included. SGLT2i significantly reduced the risk of the composite events of AF and AFL in patients with CKD (0.65% vs. 0.91%; RR 0.73, 95% CI 0.56-0.95, *P* = 0.02) in overall population, but did not reduce the risk of AF (0.56% vs. 0.75%; RR 0.76, 95% CI 0.57-1.01, *P* = 0.06) or AFL (0.097% vs. 0.17%; RR 0.58, 95% CI 0.30–1.13, *P* = 0.11). Subgroup analysis based on sample size and follow-up duration showed that SGLT2i reduced the risk of AF in trials with sample size more than 1,000 and follow-up duration longer than 2 years (0.59% vs. 0.80%; RR 0.74, 95% CI 0.55–0.99, *P* = 0.04). Subgroup analysis based on different populations showed that SGLT2i reduced the risk of AF in patients with CKD (partial without diabetes) (0.48% vs. 0.90%; RR 0.53, 95% CI 0.33–0.85, *P* = 0.009), while had no effect on AF in patients with both diabetes and CKD. Subgroup analysis based on different types of SGLT2i showed that only empagliflozin reduced the risk of AF compared to placebo (0.51% vs. 0.94%; RR 0.55, 95% CI 0.31–0.96, *P* = 0.04).

**Conclusion:**

SGLT2i could reduce the risk of the composite events of AF and AFL in patients with CKD, and also could reduce the risk of AF in trials with large sample size and long follow-up duration.

**Systematic Review Registration:**

https://www.crd.york.ac.uk/PROSPERO/view/CRD420251053244.

## Introduction

Chronic kidney disease (CKD), defined as estimated glomerular filtration rate (eGFR) < 60 mL/min/1.73 m^2^ or presence of kidney damage persisting for 3 months or more, is a hidden disease characterized by a gradual decline in renal function with or without structural changes in the kidney, and is an important cause of cardiovascular disease. According to data from the 2023 ISN Global Kidney Health Atlas (ISN-GKHA), the median global CKD prevalence rate is 9.5% (Wing-Shing Fung et al., 2024). Atrial fibrillation (AF) is the most common arrhythmia in clinical practice, which is associated with significantly increased risk of stroke, heart failure, myocardial infarction, dementia, CKD and cardiovascular mortality (Van Gelder et al., 2024; Ko et al., 2025). CKD and AF often coexist and share multiple common risk factors, such as age, male sex, cardiovascular disease, hypertension, diabetes, heart failure, and obesity (Ding et al., 2021). CKD is an independent risk factor for AF (Ruiz-García et al., 2024), and with the progression of CKD, the incidence of AF increases. The nationwide population-based study which included 4827 987 individuals without prior AF showed that the annual incidence rate of AF was 1.17 per 1,000 person-years among subjects without CKD, 1.55 for stage 1 CKD, 1.86 for stage 2 CKD, 2.1 for stage 3 CKD, and 4.33 for stage 4 CKD (Kim et al., 2023). Atrial flutter (AFL) is another common arrhythmia. Although AFL has different electrophysiological mechanism from AF, they have the similar complications. There is a bidirectional relationship between CKD and AF/AFL. Renal dysfunction can promote the initiation and maintenance of AF/AFL, while unmanageable AF/AFL accelerates the decline of renal function (Wang et al., 2022; Yoshikawa et al., 2021). Common available anti-arrhythmic drugs or catheter ablation have limited efficacy and some side effects (Donniacuo et al., 2023). We urgently hope to find more effective and safer treatment measures.

Sodium-glucose co-transporter-2 inhibitor (SGLT2i) is a novel hypoglycemic drug and works by inhibiting the re-absorption of sodium and glucose in the kidney that has been proven to have cardiac and renal protective effects (Girardi et al., 2024). Previous clinical data showed that SGLT2i may exert preventive effect against AF and AFL by inhibiting electrical remodeling, correcting metabolic disorders, and altering epigenetic networks (Donniacuo et al., 2023). Recently, some meta-analyses (Liao et al., 2024; Mariani et al., 2024; Zhang et al., 2024) evaluated the effect of SGLT2i on AF in patients with CKD, but the results are inconclusive. Meanwhile, a new trial (Herrington et al., 2023) about with field has been published. Therefore, we performed this meta-analysis to evaluate whether SGLT2i can reduce the risk of AF/AFL in patients with CKD.

## Methods

### Study search and data sources

This meta-analysis was performed according to the Preferred Reporting Items for Systematic Reviews and Meta-Analyses (PRISMA) guidelines (Page et al., 2021). An online search of PubMed, Embase, Cochrane Library, and Clinical Trials.gov were performed up to December 2024. The main search terms (“sodium-glucose transporter 2 inhibitors” OR “sodium-glucose cotransporter 2 inhibitors” OR “SGLT2i” OR “canagliflozin” OR “JNJ 28431754” OR “dapagliflozin” OR “BMS 512148” OR “empagliflozin” OR “BI 10773” OR “ertugliflozin” OR “PF04971729” OR “sotagliflozin” OR “LX4211”) and (“chronic kidney disease” OR “CKD” OR “Renal Insufficiency”) were used with no language restrictions. The search strategies are shown in Supplementary Materials S1–S4. References from selected clinical trials, recent meta-analyses and review articles were also manually searched.

### Inclusion and exclusion criteria

Eligibility criteria for included trials required: (1) randomized double-blind placebo-controlled trials; (2) comparing SGLT2i with matching placebo; (3) AF/AFL as adverse events were clearly reported in each group. Excluded criteria mainly included: (1) non-placebo control; (2) lack of information on the occurrences of AF/AFL; (3) the research subject is not CKD.

### Study selection and outcome of interest

All studies were independently screened by two authors (Q.Y. and C.W.) for article titles and abstracts to determine eligible studies. Full texts of potentially relevant reports were then reviewed by the two authors. Disagreements were addressed by discussion with a third author (Y.D.).

The primary outcome of our meta-analysis was the incidence of AF and/or AFL between SGLT2i and placebo in all included studies. Subgroup analyses were performed in different populations, different types of SGLT2i, different sample sizes and different follow-up durations.

### Data extraction and quality assessment

In our meta-analysis, the characteristics of each study (first author or the name of RCTs, year of publication, and registration number, number of patients in each group, interventions, eGFR, the follow-up duration, demographic data of patients, systolic blood pressure (SBP), diastolic blood pressure (DBP), references) were extracted into Excel. All data were obtained from Clinical trials.gov or Supplementary Material. The Cochrane tool for assessing risk of bias was used for the quality assessment of the studies.

### Statistical analysis

A fixed-effects or random-effects model was used to estimate the risk ratios (RRs) and 95% confidence intervals (CIs) depending on heterogeneity. Statistical heterogeneity was assessed using the *X* ^*2*^-test and *I* ^*2*^. The *X* ^*2*^-test *P* value greater than 0.10 and *I* ^*2*^ less than 50% represents low heterogeneity, a fixed-effect model was used, otherwise a random-effect model was used. The *P* value threshold for statistical significance was set at 0.05 for the effect sizes. Publication bias was tested using funnel plots and the Egger’s test. All statistical analyses were performed using Review manager 5.3 and Stata software 15.0.

## Results

### Characteristics of the included studies

10 RCTs with 28,712 patients were identified for inclusion in the analysis. Of all trials, 14,711 patients received SGLT2i (2 trials with canagliflozin (Yale et al., 2014; Perkovic et al., 2019), 2 with dapagliflozin (Pollock et al., 2019; Heerspink et al., 2020), 2 with empagliflozin (Barnett et al., 2014; Herrington et al., 2023), 1 with ertugliflozin (Grunberger et al., 2018) and 3 with sotagliflozin (Bhatt et al., 2021; Cherney et al., 2021; Cherney et al., 2023) and 14,001 patients received placebo. The eGFR ranged from 15 to 90 mL/min/1.73 m^2^, the mean age ranged from 61.8 to 69.5 years and the follow-up duration ranged from 28 weeks to 4.6 years. The baseline characteristics of the studies included are shown in Table 1.

**TABLE 1:** Baseline characteristics of all trials included in the meta-analysis.

Study, year	NCT number	Number of patients SGLT2i/placebo	Mean age (year)	Male (%)	Population	eGFR (ml/min/1.73m^2^)	Follow-up duration	Interventions	References
EMPA-REG RENAL, 2014	NCT01164501	738 419/319	63.9	58.3	T2DM and CKD	NA	65 weeks	Empagliflozin 10 and 25 mg	Barnett et al. (2014)
Yale et al. (2014)	NCT01064414	269 179/90	68.5	60.6	T2DM and CKD	39.4 ± 6.9	52 weeks	Canagliflozin 100 and 300 mg	Yale et al. (2014)
VERTIS RENAL, 2018	NCT01986855	467 313/154	67.3	49.5	T2DM and CKD	46.6 ± 8.8	54 weeks	Ertugliflozin 5 and 15 mg	Grunberger et al. (2018)
CREDENCE, 2019	NCT02065791	4,401 2,200/2,197	63	66.1	T2DM and CKD	56.2 ± 18.2	4.6 years	Canagliflozin 100 mg	Perkovic et al. (2019)
DELIGHT, 2019	NCT02547935	293 145/148	64.4	70.8	T2DM and CKD	NA	28 weeks	Dapagliflozin 10 mg	Pollock et al. (2019)
DAPA-CKD, 2020	NCT03036150	4,298 2,149/2,149	61.8	66.9	CKD	43.1 ± 12.3	39.2 months	Dapagliflozin 10 mg	Heerspink et al. (2020)
SCORED, 2021	NCT03315143	10,577 5,291/5,286	68.3	55.1	T2DM and CKD	44.3 ± 10.7	30 months	Sotagliflozin 200 mg	Bhatt et al. (2021)
SOTA-CKD4, 2021	NCT03242018	277 184/93	67.4	48.7	T2DM and CKD	24.0 ± 4.0	60 weeks	Sotagliflozin 200 and 400 mg	Cherney et al. (2021)
EMPA-KIDNEY, 2023	NCT03594110	6,609 3,304/3,305	63.3	66.8	CKD	37.3 ± 14.5	39 months	Empagliflozin 10 mg	Herrington et al. (2023)
SOTA-CKD3, 2023	NCT03242252	787 527/260	69.5	56.4	T2DM and CKD	45.0 ± 8.1	60 weeks	Sotagliflozin 200 and 400 mg	Cherney et al. (2023)

SGLT2i, sodium-glucose co-transporter-2, inhibitors; T2DM, type 2 diabetes mellitus; CKD, chronic kidney disease; eGFR, estimated glomerular filtration rate; NA, not available.

**FIGURE 1:**
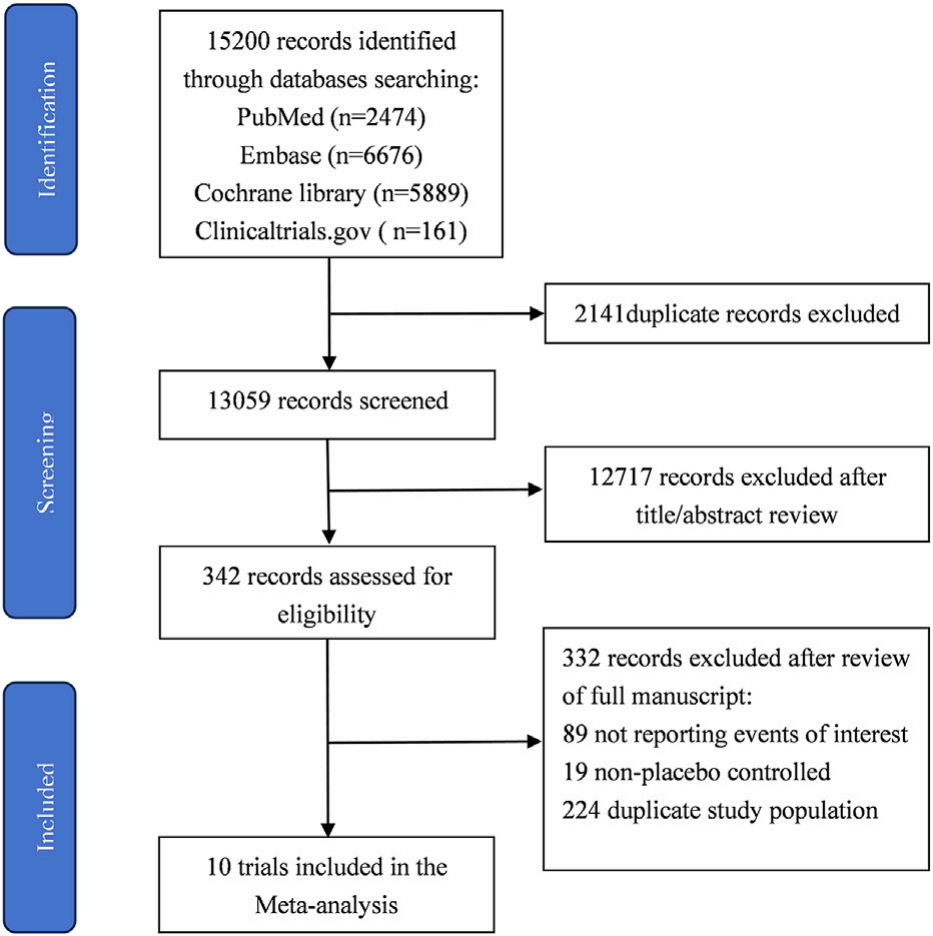
The literature screening diagram.

The process of study selection is summarized in Figure 1. The quality assessment of the included RCTs according to the Cochrane Risk of Bias Tool was presented in Supplementary Material S5. All RCTs were considered with high methodological quality. Publication bias was evaluated using Egger’s test. The results suggested no significant risk of publication bias existed in overall population (AF and AFL: *P* = 0.718; AF: *P* = 0.547; AFL: *P* = 0.624).

### Overall efficacy outcomes

**FIGURE 2:**
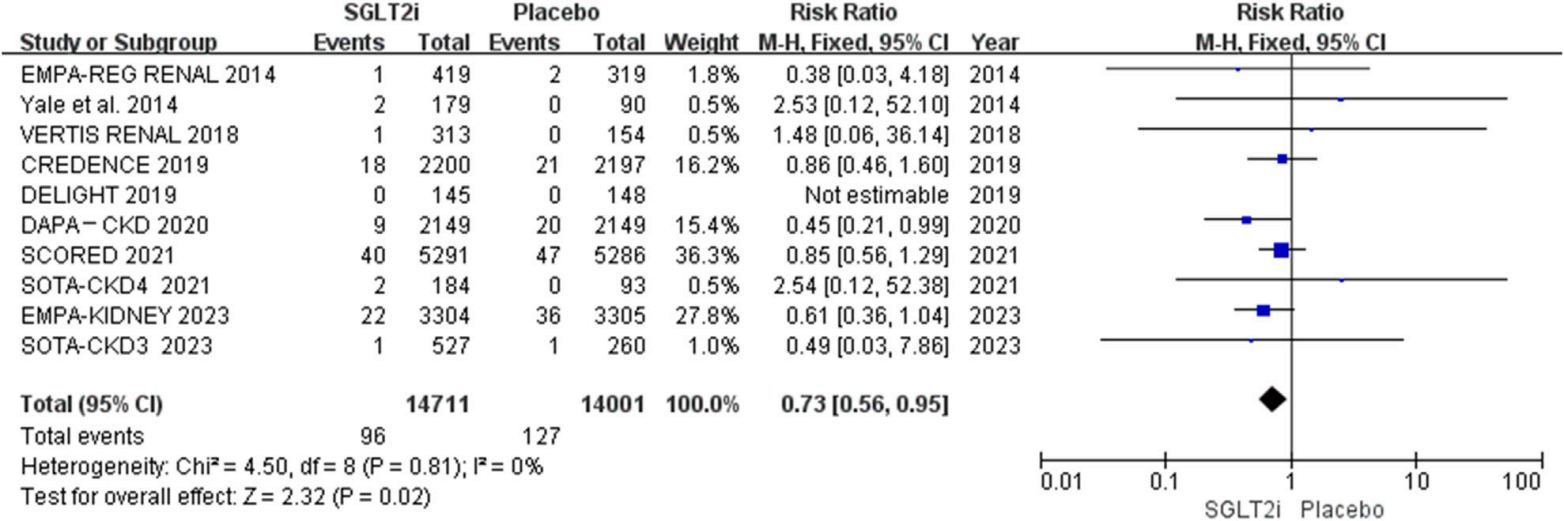
Effect of SGLT2i on the composite events of AF and AFL in the patients with CKD.

AF and AFL occurred in 96 of 14,711 patients in the SGLT2i group and 127 of 14,001 patients in the placebo group. The meta-analysis showed that SGLT2i significantly reduced the risk of the composite events of AF and AFL in patients with CKD (0.65% vs. 0.91%; RR 0.73, 95% CI 0.56-0.95, *P* = 0.02), and there was no substantial heterogeneity (*P* = 0.81, *I* ^2^ = 0%). The results are shown in Figure 2.

**FIGURE 3:**
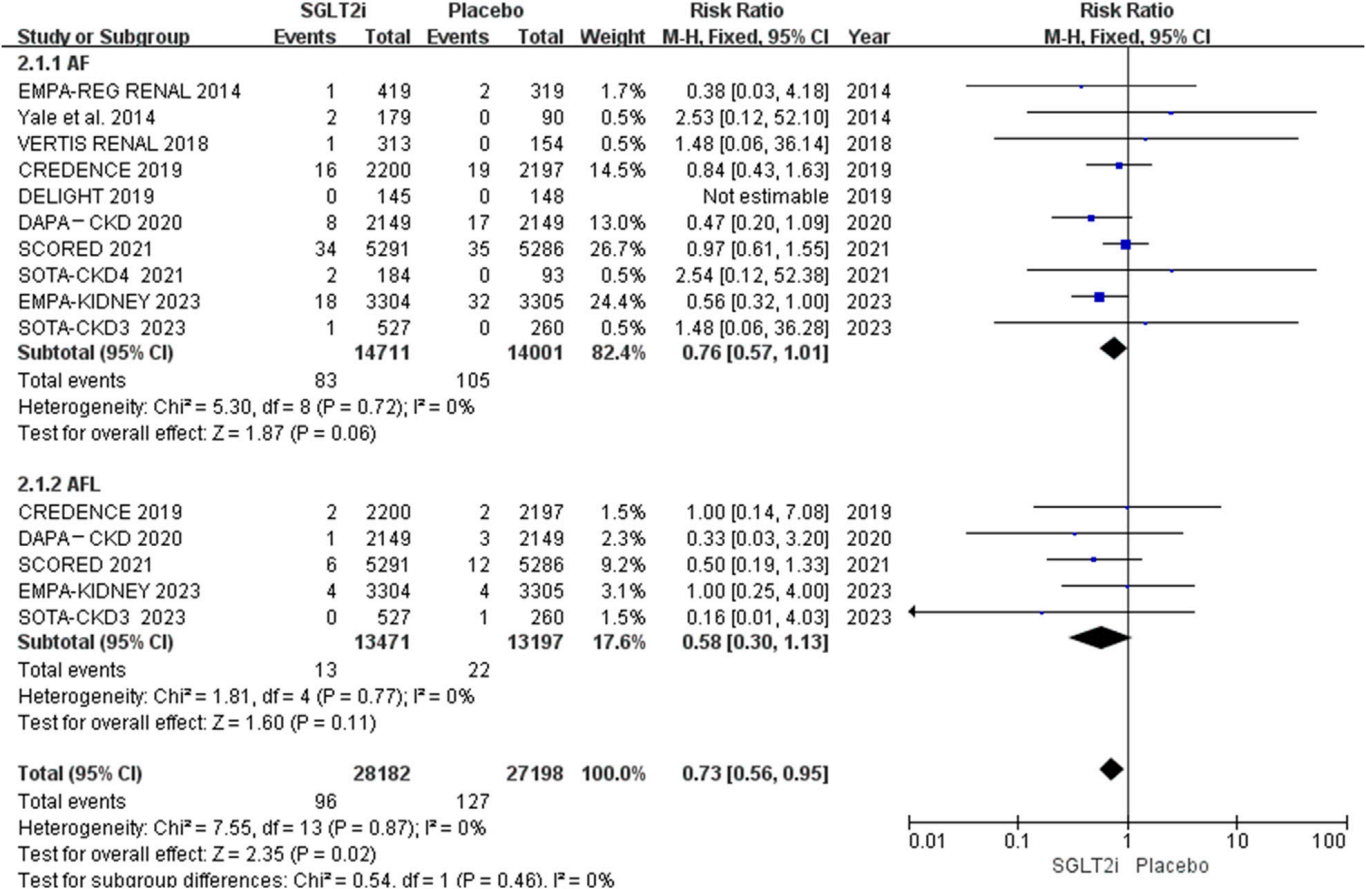
Effect of SGLT2i on AF or AFL in the patients with CKD.

Of the 10 RCTs, 10 reported AF events, and 5 reported AFL events (Perkovic et al., 2019; Heerspink et al., 2020; Bhatt et al., 2021; Cherney et al., 2023; Herrington et al., 2023). AF occurred in 83 of 14,711 patients in the SGLT2i group and 105 of 14,001 patients in the placebo group. AFL occurred in 13 of 13,471 patients in the SGLT2i group and 22 of 13,197 patients in the placebo group. The meta-analysis showed that SGLT2i did not reduce the risks of neither AF (0.56% vs. 0.75%; RR 0.76, 95% CI 0.57–1.01, *P* = 0.06) nor AFL (0.097% vs. 0.17%; RR 0.58, 95% CI 0.30-1.13, *P* = 0.11) in patients with CKD. The results are shown in Figure 3. However, the risk of AF between these two groups was borderline significant. The funnel plot comparing the incidence of AF and AFL between these two groups revealed no apparent asymmetry upon visual inspection. The results are shown in Supplementary Material S6.

### Subgroup outcome

**FIGURE 4:**
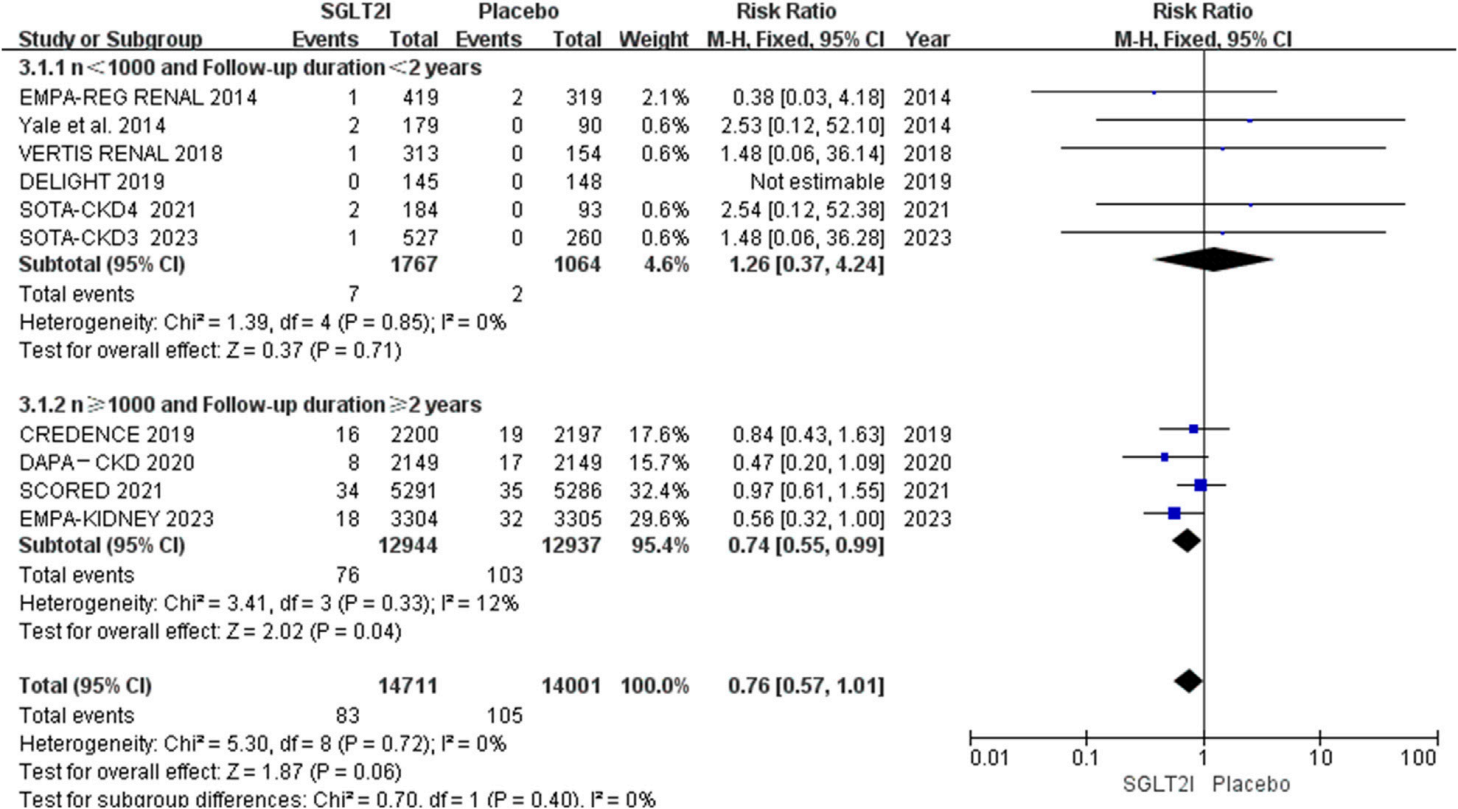
Subgroup analysis of AF based on sample size and follow-up duration.

Of the 10 RCTs, 4 trials (Perkovic et al., 2019; Heerspink et al., 2020; Bhatt et al., 2021; Herrington et al., 2023) had more than 1,000 sample size and longer than 2 years follow-up duration, which contained 90% of the overall patients. Subgroup analysis of AF events based on these showed that 76 of 12,944 patients in the SGLT2i group in comparison to a higher incidence of 103 out of 12,937 for the placebo group, SGLT2i could reduce the incidence of AF in trials with large sample size and long follow-up duration (0.59% vs. 0.80%; RR 0.74, 95% CI 0.55-0.99, *P* = 0.04). The results are shown in Figure 4.

**FIGURE 5:**
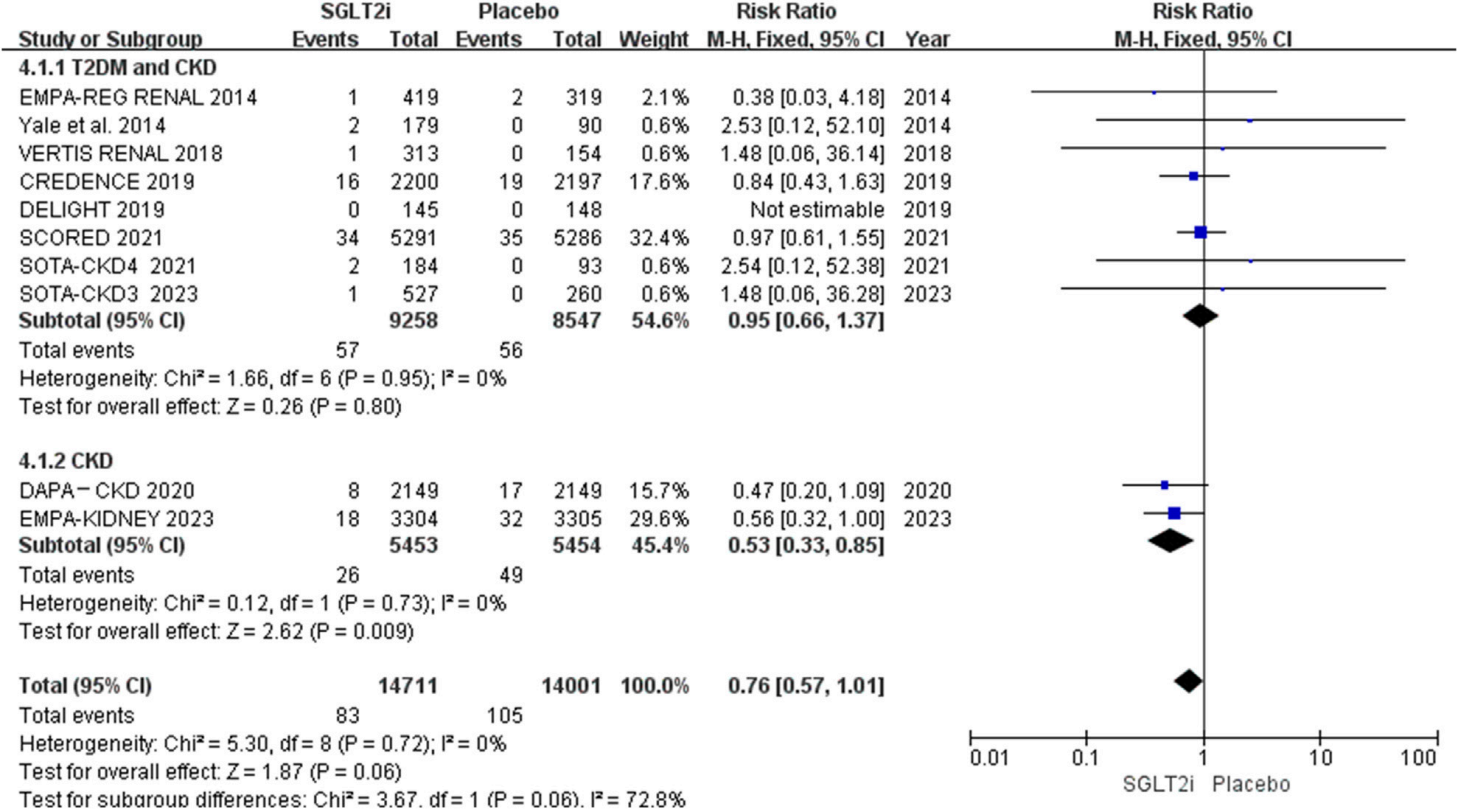
Subgroup analysis of AF based on different populations.

Subgroup analysis of AF events was performed based on different populations. 8 trials enrolled 17,805 patients with both T2DM and CKD, and 2 trials (DAPA-CKD 2020, EMPA-KIDNEY 2023) enrolled 10,907 patients with CKD. Approximately 54% participants of EMPA-KIDNEY and 33% of DAPA-CKD do not have diabetes. The results showed that SGLT2i had effect on reducing the risk of AF in patients with CKD (partial without diabetes) (0.48% vs. 0.90%; RR 0.53, 95% CI 0.33-0.85, *P* = 0.009), and had no effect in patients with both T2DM and CKD (0.62% vs. 0.66%; RR 0.95, 95% CI 0.66-1.37, *P* = 0.79). The results are shown in Figure 5.

**FIGURE 6:**
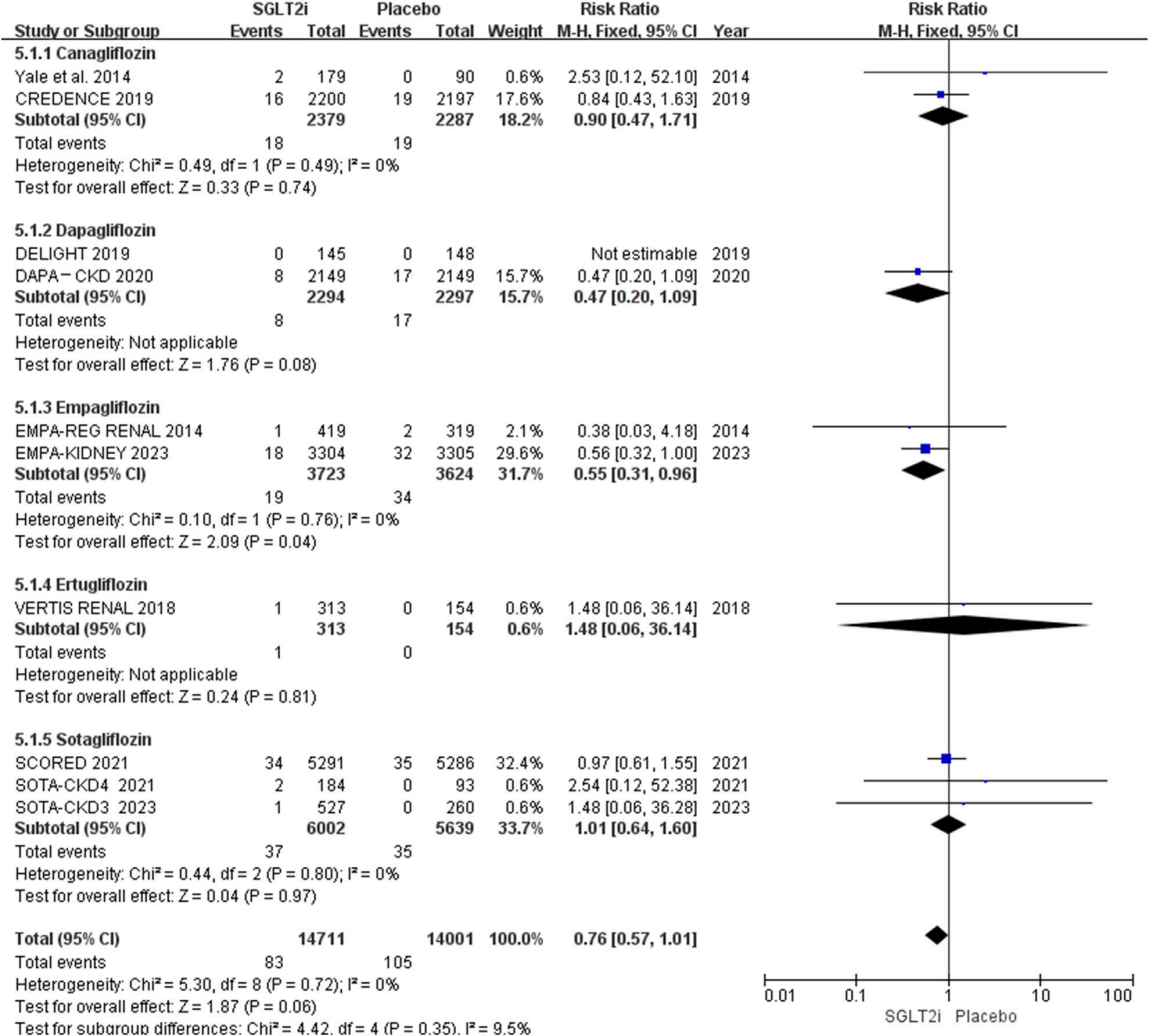
Subgroup analysis of AF based on different types of SGLT2i.

Subgroup analysis based on different types of SGLT2i showed that only empagliflozin could reduce the risk of AF (0.51% vs. 0.94%; RR 0.55, 95% CI 0.31-0.96, *P* = 0.04), while canagliflozin (0.76% vs. 0.83%; RR 0.90, 95% CI 0.47-1.71, *P* = 0.74), dapagliflozin (0.35% vs. 0.74%; RR 0.47, 95% CI 0.20-1.09, *P* = 0.08), ertugliflozin (0.32% vs. 0.00%; RR 1.48, 95% CI 0.06-36.14, *P* = 0.81) and sotagliflozin (0.62% vs. 0.62%; RR 1.01, 95% CI 0.64-1.60, *P* = 0.97) had no effect in patients with CKD. The results are shown in Figure 6.

## Discussion

The anti-arrhythmic drugs for the treatment of AF and AFL remains a global research challenge. SGLT2i as a rising star in the medical field, has been extensively studied for its preventive effect against AF through various pathways, but the results are inconclusive. In recent years, some meta-analyses have been published looking at the relationship between SGLT2i and AF/AFL, but none of them specifically focused on patients with CKD. Meanwhile, a new large RCT studying the preventive effect of Empagliflozin on AF (EMPA-KIDNEY) (Herrington et al., 2023) was included in our meta-analysis. This trial included 6,609 patients with CKD, and followed for a median of 39 months. The data showed that empagliflozin decreased the incidence of reported episodes of AF/AFL adverse events. Therefore, we conducted this meta-analysis and hoped to provide more options for the treatment of AF and AFL in patients with CKD.

As is well known, AF and AFL are common atrial arrhythmia. Although they have different electrophysiological mechanisms, both of them can cause rapid ventricular rate and atrial thrombosis, and lead to similar complications and increase mortality. Therefore, they are often analyzed together (Li et al., 2021; Liao et al., 2024). Our meta-analysis evaluated AF and AFL events in 28,712 patients with CKD who received SGLT2i (canagliflozin, dapagliflozin, empagliflozin, ertugliflozin and sotagliflozin) or placebo. The results showed that SGLT2i did not reduce the risks of neither AF (0.56% vs. 0.75%; RR 0.76, 95% CI 0.57-1.01, *P* = 0.06) nor AFL (0.097% vs. 0.17%; RR 0.58, 95% CI 0.30–1.13, *P* = 0.11), but there were potentially effective trends of SGLT2i on preventing AF and AFL, especially on AF. When AF and AFL were analyzed together, the results showed that SGLT2i reduced the composite events of AF and AFL by 28.57% compared with placebo (0.65% vs. 0.91%), the preventive effect emerges.

Previous Zhang et al.‘s meta-analysis (Zhang et al., 2024) showed that SGLT2i did not decrease the risk of AF, regardless of follow-up duration, type or dose of the drug, or the patient population. Their analysis incorporated data from 101,100 patients sourced from 46 trials, and included 9 trials with CKD (8 with both DM and CKD (Barnett et al., 2014; Yale et al., 2014; Grunberger et al., 2018; Perkovic et al., 2019; Pollock et al., 2019; Bhatt et al., 2021; Cherney et al., 2021; Cherney et al., 2023) and 1 with CKD only (Heerspink et al., 2020)). Subgroup analysis suggested that in patients with CKD (n = 22,109), AF was not prevented by SGLT2i compared with placebo (RR 0.84, 95% CI 0.61-1.18, *P* = 0.793) (Zhang et al., 2024). However, our analysis suggested although SGLT2i did not reduce the occurrence of AF in the overall population. In the subgroup analysis, SGLT2i could reduce the risk of AF by 26.25% compared with placebo (0.59% vs. 0.80%) in trials with large sample size (n ≥ 1,000), long follow-up (≥2 years). The incidence of AF in our meta-analysis is only 6.5‰, so large sample size and long follow-up are essential for trials to evaluate the difference of the incidences between the SGLT2i and placebo groups.

When we conducted subgroup analysis on the study population, we found that SGLT2i could not prevent the risk of AF in patients with both CKD and T2DM, but could prevent the risk of AF in patients with CKD. Although only two trials were included in CKD subgroup, both DAPA-CKD (Heerspink et al., 2020) and EMPA-KIDNEY (Herrington et al., 2023) trials had large sample sizes and long follow-up duration. Participants of these two trials were not all non-diabetic patients. Approximately 54% participants of EMPA-KIDNEY and 33% of DAPA-CKD do not have diabetes. Subgroup analysis showed that SGLT2i reduced the risk of AF by 46.67% compared with placebo (0.48% vs. 0.90%). Perhaps SGLT2i played preventive effect on AF/AFL in these non-diabetic patients, this potential effect and mechanism need further researches.

When we conducted subgroup analysis on the different types of SGLT2i we found that only empagliflozin could reduce the risk of AF. This result is not consistent with previous meta-analyses, which showed that all types of SGLT2i did not decrease the risk of AF (Zhang et al., 2024), or only dapagliflozin lowered the risk of AF (Liao et al., 2024). Perhaps because a new large RCT of empagliflozin (EMPA-KIDNEY) was included, and our study only focuses on CKD patients. It must be mentioned that there were only two trials of empagliflozin (Barnett et al., 2014; Herrington et al., 2023) included, and one (Barnett et al., 2014) of them had small sample size. Therefore, further researches are needed to evaluate whether empagliflozin can reduce the risk of AF in patients with CKD.

It should be noted that AF and AFL are both reported as adverse events rather than primary or secondary outcomes in all included RCTs of our meta-analysis. If patients with paroxysmal AF/AFL had no symptoms, or had symptoms but happened to be in sinus rhythm when electrocardiograms were recorded, AF/AFL events would not be reported in these patients. Therefore, the incidence data of AF and AFL are likely to be underestimated. In the future, with the use of smartwatches in combination with electrocardiograms and Holter for AF/AFL monitoring, this problem will be solved.

At present, the protective mechanism of SGLT2i against AF/AFL with CKD is not very clear. It may work through the following aspects. First, patients with CKD commonly exhibit chronic systemic inflammation syndrome characterized by increased levels of IL-1β, IL-6, and C-reactive protein (CRP) (Tinti et al., 2021). Chronic systemic inflammation can lead to atrial electrical remodeling, fibrosis, hypertrophy, and cell apoptosis, thereby participate in the occurrence of AF (Harada and Nattel, 2021; Hu et al., 2023). Meanwhile, increasing evidence indicated that inflammasome signaling in atrial cardiomyocytes caused inflammation and contributed to AF pathogenesis (Ajoolabady et al., 2022), and CKD could induce pro-inflammatory milieu, enhance atrial inflammasome activity (Song et al., 2023). SGLT2i can inhibit inflammatory responses, including modulation of the NLRP3 inflammasome, reduction of pro-inflammatory markers and reduction of oxidative stress (Zelniker et al., 2020). Second, SGLT2i not only increase glucose excretion in urine and lower blood glucose levels, but also produce natriuretic and diuretic effects, which ultimately lead to a decrease in atrial volume (Aguilar-Gallardo et al., 2022). Third, SGLT2i could improve mitochondrial function and enhance cardiac energy status by increasing ketone oxidation and cardiomyocyte Na^+^/H^+^ exchange (Lan et al., 2021; Thirumathyam et al., 2022; Koizumi et al., 2023).

## Limitations

There are several limitations in our meta-analysis. First, different stages of CKD based on eGFR may have different responses to SGLT2i, but we cannot estimate detailed data of AF/AFL events in different stages of CKD. Second, all trials recorded the AF/AFL events as adverse events rather than primary or secondary outcomes, which may lead to incomplete data. Third, some of the studies we included used different doses of SGLT2i, and further researches are needed to evaluate if there is a dose-effect relationship.

## Conclusion

In our analysis, SGLT2i could significantly reduce the risk of AF/AFL in overall population with CKD, and the risk of AF in trials with large sample size and long follow-up duration.

## Data Availability

The datasets presented in this study can be found in online repositories. The names of the repository/repositories and accession number(s) can be found in the article/Supplementary Material.
