# Supplementary material for: The preventive effect of sodium-glucose co-transporter-2 inhibitors on atrial fibrillation and atrial flutter in patients with chronic kidney disease: a meta-analysis
Source: Front Pharmacol. 2025 May 20;16:1585491. doi: 10.3389/fphar.2025.1585491 (PMC12129918; doi:10.3389/fphar.2025.1585491)
Supplement: Supplementary file 1 [file Supplementaryfile1.pdf]

Supplementary File

- Supplement 1. Search strategy of PubMed
- Supplement 2. Search strategy of Cochrane Library
- Supplement 3. Search strategy of Embase
- Supplement 4. Search strategy of Clinical Trials.gov
- Supplement 5. Quality assessment of the included RCTs.
- Supplement 6. Funnel plot of impact of SGLT2i on AF and AFL.
- Supplement 7. Baseline characteristics (blood pressure) of all trials included in the meta-analysis.

Supplement 1. Search strategy of PubMed

Intervention	#1	‘Sodium-Glucose Transporter 2 Inhibitors’ OR ‘Sodium-Glucose Transporter 2 Inhibitor’ OR ‘Sodium Glucose Transporter 2 Inhibitors’ OR ‘Sodium Glucose Transporter 2 Inhibitor’ OR ‘SGLT-2 Inhibitors’ OR ‘SGLT-2 Inhibitor’ OR ‘SGLT2 Inhibitors’ OR ‘SGLT2 Inhibitor’ OR ‘Inhibitor, SGLT-2’ OR ‘Inhibitor, SGLT2’ OR ‘Gliflozins’ OR ‘Gliflozin’
	#2	‘Canagliflozin’ OR ‘invokana’ OR ‘TA7284’ OR ‘JNJ28431754’
	#3	‘Dapagliflozin’ OR ‘farxiga’ OR ‘forxiga’ OR ‘BMS512148’
	#4	‘Empagliflozin’ OR ‘jardiance’ OR ‘BI10773’
	#5	‘Ertugliflozin’ OR ‘PF04971729’
	#6	‘Sotagliflozin’
	#7	#1 OR #2 OR #3 OR #4 OR #5 OR #6
Patient population	#8	‘Renal Insufficiencies, Chronic’ OR ‘Chronic Kidney Insufficiency’ OR ‘Chronic Kidney Insufficiencies’ OR ‘Kidney Insufficiencies, Chronic’ OR ‘Chronic Renal Insufficiency’ OR ‘Kidney Insufficiency, Chronic’ OR ‘Chronic Kidney Diseases’ OR ‘Chronic Kidney Disease’ OR ‘Disease, Chronic Kidney’ OR ‘Diseases, Chronic Kidney’ OR ‘Kidney Disease, Chronic’ OR ‘Kidney Diseases, Chronic’ OR ‘Chronic Renal Diseases’ OR ‘Chronic Renal Disease’ OR ‘Disease, Chronic Renal’ OR ‘Diseases, Chronic Renal’ OR ‘Renal Disease, Chronic’ OR ‘Renal Diseases, Chronic’
Type of study	#9	‘randomized controlled trial’ OR ‘RCT’ OR ‘controlled clinical trial’ OR ‘randomized’ OR ‘placebo’
Combined	#10	#7 AND #8 AND #9

Supplement 2. Search strategy of Cochrane Library

Intervention	#1	‘Sodium-Glucose Transporter 2 Inhibitors’ OR ‘Sodium Glucose Transporter 2 Inhibitors’ OR ‘Sodium Glucose Transporter 2 Inhibitor’ OR ‘Sodium-Glucose Transporter 2 Inhibitor’ OR ‘SGLT-2 Inhibitors’ OR ‘SGLT 2 Inhibitor’ OR ‘Inhibitor, SGLT-2’ OR ‘SGLT-2 Inhibitor’ OR ‘SGLT 2 Inhibitors’ OR ‘SGLT2 Inhibitor’ OR ‘Inhibitor, SGLT2’ OR ‘SGLT2 Inhibitors’ OR ‘Gliflozin’ OR ‘Gliflozins’
	#2	‘Canagliflozin’ OR ‘invokana’ OR ‘canagliflozin hemihydrate’ OR ‘TA7284’ OR ‘JNJ28431754’
	#3	‘Dapagliflozin’ OR ‘farxiga’ OR ‘forxiga’ OR ‘BMS512148’
	#4	‘Empagliflozin’ OR ‘jardiance’ OR ‘BI10773’
	#5	‘Ertugliflozin’ OR ‘PF04971729’
	#6	‘Sotagliflozin’ OR ‘LX4221’
	#7	#1 OR #2 OR #3 OR #4 OR #5 OR #6
Patient population	#8	‘Renal Insufficiency, Chronic’ OR ‘Kidney Insufficiencies, Chronic’ OR ‘Chronic Kidney Insufficiency’ OR ‘Renal Insufficiencies, Chronic’ OR ‘Kidney Insufficiency, Chronic’ OR ‘Chronic Renal Insufficiencies’ OR ‘Chronic Renal Insufficiency’ OR ‘Chronic Kidney Insufficiencies’ OR ‘Renal Disease, Chronic’ OR ‘Kidney Disease, Chronic’ OR ‘Disease, Chronic Kidney’ OR ‘Chronic Kidney Diseases’ OR ‘Chronic Kidney Disease’ OR ‘Disease, Chronic Renal’ OR ‘Renal Diseases, Chronic’ OR ‘Diseases, Chronic Kidney’ OR ‘Chronic Renal Diseases’ OR ‘Kidney Diseases, Chronic’ OR ‘Diseases, Chronic Renal’ OR ‘Chronic Renal Disease’
Type of study	#9	‘randomized controlled trial’ OR ‘RCT’ OR ‘controlled clinical trial’ OR ‘randomized’ OR ‘placebo’
Combined	#10	#7 AND #8 AND #9

Supplement 3. Search strategy of Embase

Intervention	#1	'gliflozin' OR 'gliflozin derivative' OR 'gliflozins' OR 'SGLT2 inhibitor' OR 'SGLT2 inhibitors' OR 'sodium dependent glucose cotransporter 2 inhibitor' OR 'sodium glucose co-transporter 2 inhibitor' OR 'sodium-glucose transporter 2 inhibitors' OR 'SGLT2i' OR 'sodium glucose cotransporter 2 inhibitor'
	#2	'Canagliflozin' OR 'invokana' OR 'sulisent' OR 'canagliflozin hemihydrate' OR 'TA7284' OR 'JNJ28431754'
	#3	'Dapagliflozin' OR 'farxiga' OR 'forxiga' OR 'BMS512148'
	#4	'Empagliflozin' OR 'jardiance' OR 'BI10773'
	#5	'Ertugliflozin' OR 'PF04971729'
	#6	'Sotagliflozin' OR 'LX4221'
	#7	#1 OR #2 OR #3 OR #4 OR #5 OR #6
Patient population	#8	'Chronic Kidney Disease' OR 'chronic kidney disorder' OR 'chronic kidney insufficiency' OR 'chronic nephropathy' OR 'chronic renal disease' OR 'chronic renal failure' OR 'chronic renal insufficiency' OR 'kidney chronic failure' OR 'kidney disease chronic' OR 'kidney failure, chronic' OR 'kidney function, chronic disease' OR 'renal insufficiency, chronic' OR 'chronic kidney failure'
Type of study	#9	'randomized controlled trial' OR 'RCT' OR 'controlled clinical trial' OR 'randomized' OR 'placebo'
Combined	#10	#7 AND #8 AND #9

Supplement 4. Search strategy of Clinical Trials.gov

Intervention	#1	‘sodium-glucose transporter 2 inhibitors’ OR ‘sodium-glucose cotransporter 2 inhibitors’ OR ‘SGLT2i’ OR ‘canagliflozin’ OR ‘JNJ 28431754’ OR ‘dapagliflozin’ OR ‘BMS 512148’ OR ‘empagliflozin’ OR ‘BI 10773’ OR ‘ertugliflozin’ OR ‘PF04971729’ OR ‘sotagliflozin’ OR ‘LX4211’
Patient population	#2	‘Chronic Kidney Disease’ OR ‘CKD’ OR ‘Renal Insufficiency’
Combined	#3	#1 AND #2

Supplement 5. Quality assessment of the included RCTs.

Study	Random sequence generation	Allocation concealment	Blinding in performance	Blinding in outcome detection	Incomplete outcome data	Reporting bias	Other bias
EMPA-REG RENAL, 2014	Low risk	Low risk	Low risk	Low risk	Low risk	Low risk	Low risk
Yale et al., 2014	Low risk	Low risk	Low risk	Unclear risk	Low risk	Low risk	Low risk
VERTIS RENAL, 2018	Low risk	Low risk	Low risk	Low risk	Low risk	Low risk	Low risk
CREDENCE, 2019	Low risk	Low risk	Low risk	Low risk	Low risk	Low risk	Low risk
DELIGHT, 2019	Low risk	Low risk	Low risk	Unclear risk	Low risk	Low risk	Low risk
DAPA-CKD, 2020	Low risk	Low risk	Low risk	Low risk	Low risk	Low risk	Low risk
SCORED, 2021	Low risk	Low risk	Low risk	Low risk	Low risk	Low risk	Low risk
SOTA-CKD4, 2021	Low risk	Low risk	Low risk	Low risk	Low risk	Low risk	Low risk
EMPA-KIDNEY, 2023	Low risk	Low risk	Low risk	Low risk	Low risk	Low risk	Low risk
SOTA-CKD3, 2023	Low risk	Low risk	Low risk	Low risk	Low risk	Low risk	Low risk

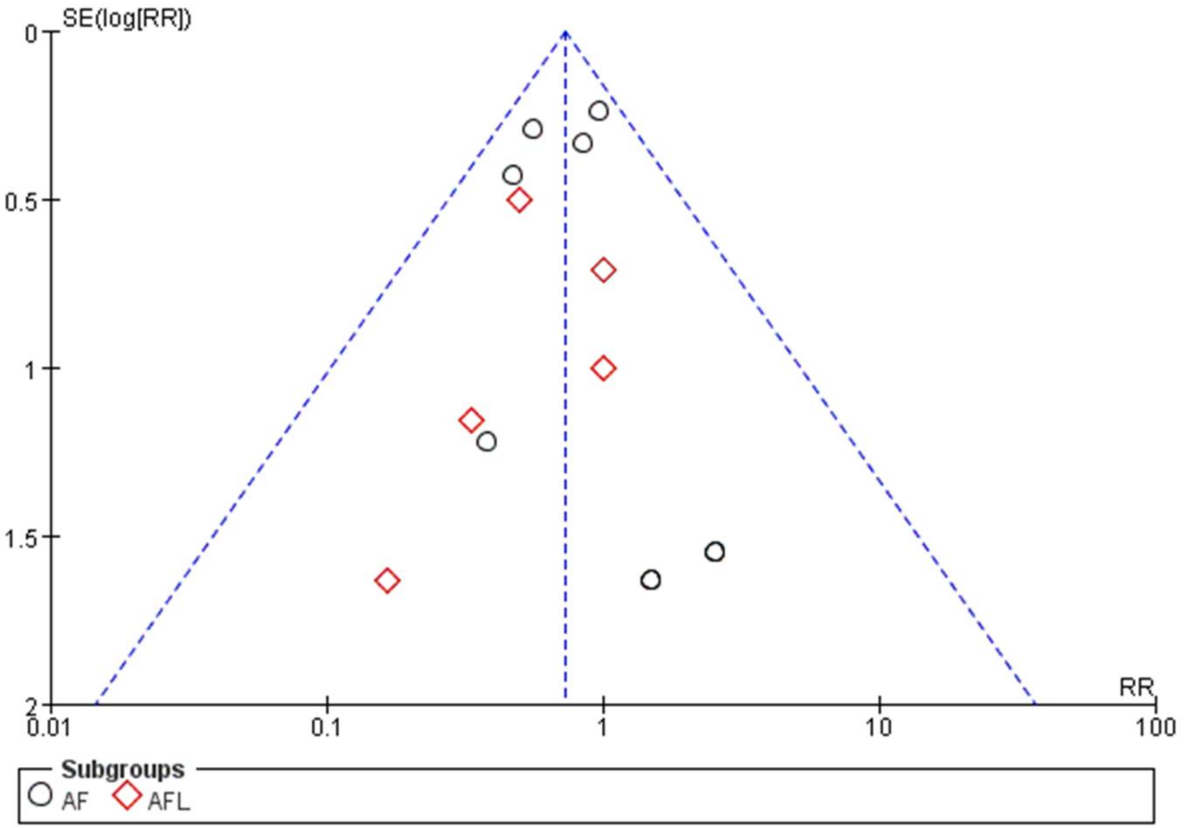

Supplement 6. Funnel plot of impact of SGLT2i on AF and AFL.

Supplement 7. Baseline characteristics（blood pressure）of all trials included in the meta-analysis.

Study,year	NCT number	SBP(mmHg) SGLT2i/placebo or overall*	DBP(mmHg) SGLT2i/placebo or overall*	References
EMPA-REG RENAL, 2014	NCT01164501	NA	NA	Barnett et al., 2014
Yale et al., 2014	NCT01064414	NA	NA	Yale et al., 2014
VERTIS RENAL, 2018	NCT01986855	NA	NA	Grunberger et al., 2018
CREDENCE, 2019	NCT02065791	140.0±15.6	78.3±9.4	Perkovic et al., 2019
DELIGHT, 2019	NCT02547935	138.0±16.5 / 140.2±18.6	76.9±9.5 / 75.7±11.5	Pollock et al., 2019
DAPA-CKD, 2020	NCT03036150	136.7±17.5 / 137.4±17.3	77.5±10.7 / 77.5±10.3	Heerspink et al., 2020
SCORED, 2021	NCT03315143	138 (127–149) / 139 (127–149)	78 (70–85) / 78 (70–85)	Bhatt et al., 2021
SOTA-CKD4, 2021	NCT03242018	144.0±15.2	NA	Cherney et al., 2021
EMPA-KIDNEY, 2023	NCT03594110	136.4±18.1 / 136.7±18.4	78.1±11.7 / 78.1±11.9	Herrington et al., 2023
SOTA-CKD3, 2023	NCT03242252	140.9±14.9	NA	Cherney et al., 2023

SGLT2i, sodium-glucose co-transporter-2 inhibitors; SBP, systolic blood pressure; DBP, diastolic blood pressure; NA, not available.
*The baseline blood pressure of SGLT2i and placebo groups was extracted; if there were no blood pressure data of the two groups, the baseline blood pressure of overall populations was extracted.
